# Semi-Supervised Encrypted Malicious Traffic Detection Based on Multimodal Traffic Characteristics

**DOI:** 10.3390/s24206507

**Published:** 2024-10-10

**Authors:** Ming Liu, Qichao Yang, Wenqing Wang, Shengli Liu

**Affiliations:** Information Engineering University, Zhengzhou 450001, China; lm_puree@outlook.com (M.L.); yangqichaoo@foxmail.com (Q.Y.); wenqingww@126.com (W.W.)

**Keywords:** encrypted malicious traffic detection, semi-supervised learning, multimodal features, network security

## Abstract

The exponential growth of encrypted network traffic poses significant challenges for detecting malicious activities online. The scale of emerging malicious traffic is significantly smaller than that of normal traffic, and the imbalanced data distribution poses challenges for detection. However, most existing methods rely on single-category features for classification, which struggle to detect covert malicious traffic behaviors. In this paper, we introduce a novel semi-supervised approach to identify malicious traffic by leveraging multimodal traffic characteristics. By integrating the sequence and topological information inherent in the traffic, we achieve a multifaceted representation of encrypted traffic. We design two independent neural networks to learn the corresponding sequence and topological features from the traffic. This dual-feature extraction enhances the model’s robustness in detecting anomalies within encrypted traffic. The model is trained using a joint strategy that minimizes both the reconstruction error from the autoencoder and the classification loss, allowing it to effectively utilize limited labeled data alongside a large amount of unlabeled data. A confidence-estimation module enhances the classifier’s ability to detect unknown attacks. Finally, our method is evaluated on two benchmark datasets, UNSW-NB15 and CICIDS2017, under various scenarios, including different training set label ratios and the presence of unknown attacks. Our model outperforms other models by 3.49% and 5.69% in F1 score at labeling rates of 1% and 0.1%, respectively.

## 1. Introduction

Network traffic serves as the medium for the transmission and exchange of information online. It contains vast amounts of valuable data. Many malicious actors intercept and alter this traffic to achieve illegal objectives. Moreover, they use network traffic to launch attacks, which often carry viruses, worms, and Trojans, posing significant risks to network security. To safeguard privacy and data security, encryption technologies are widely employed in network data transmission. The use of traffic-encryption provides a secure data-transfer channel for legitimate users, ensuring the safety of data transmission. Nonetheless, while traffic-encryption technologies protect privacy and communication security, they also introduce new security risks. Some malicious actors use encrypted channels to transmit malicious data, aiming to conceal their malicious intent and evade firewall detection, thus creating potential risks to network security.

**Figure 1:**
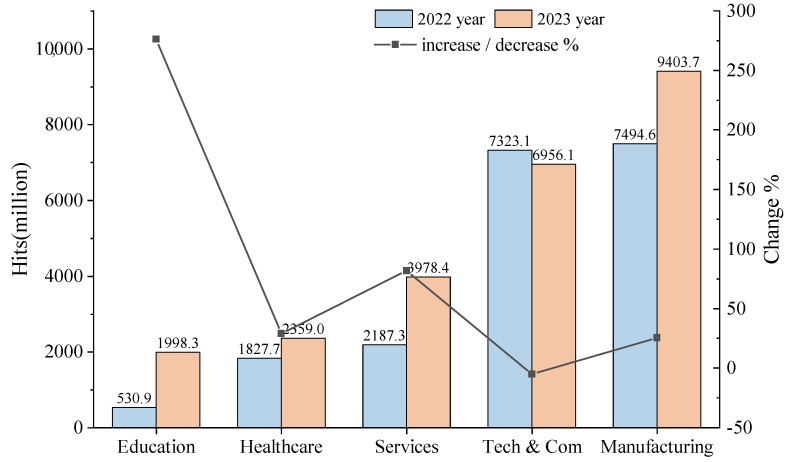
The top five industries globally affected by encrypted attacks from 2022 to 2023. The horizontal axis represents five distinct industries. The line chart shows their growth rates.

In the year 2023, according to a report [1] by Sonicwall, there was a substantial surge in browser-based exploits and adware-infested websites, with increases of 297.1% and 290.5%, respectively. This highlights a worrisome pattern where encrypted pathways are being leveraged to target weaknesses in Internet browsers and propagate spyware. Additionally, Zscaler’s 2023 report [2] on the state of encrypted attacks indicated that an alarming 85.9% of all detected threats were transmitted via encrypted channels. This emphasizes the critical necessity for comprehensive traffic scrutiny to ensure security. Figure 1 shows the top five most attacked industries and the year-over-year growth rate of attack frequency. Consequently, rapidly and accurately identifying malicious traffic within SSL/TLS encrypted flows is of paramount importance for ensuring the security of network traffic.

The core difference between identifying encrypted and unencrypted traffic lies in the alteration of distinguishing features due to encryption. When the content of data packets is encrypted, the original text is transformed into an unreadable format. As a result, various statistical characteristics at both the packet and flow levels can be altered post-encryption. This includes metrics like the total byte count of a flow, the size of individual packets, and the time gaps between the arrival of successive packets. The alterations in these features pose three key challenges to the methods of traffic detection in real-world networks. (1) Encrypted traffic-classification models have limited expressive power, and reliance on a single feature is not suitable for model generalization across multiple scenarios. Simple statistical features and TLS characteristics, which exclude inter-host interaction data, are insufficient for abstracting critical patterns of advanced attack behaviors. (2) There is a severe imbalance in the samples. Typically, in training datasets, the number of normal traffic instances significantly exceeds the number of attack samples, leading to the frequent inaccurate detection of minority classes. This extreme class imbalance has always been a significant challenge for detection problems. (3) There is an inability to detect unknown attacks. Real-world network environments are often complex and dynamic, with new types of network attacks continuously emerging. Consequently, it is common to encounter attack types during testing that were not present in the training data, and classifiers often lack the ability to recognize these, resulting in poor detection accuracy. These challenges render some traditional identification methods difficult or even inapplicable [3]. Effectively detecting and handling malicious traffic within encrypted flows is a crucial measure for defending against network attacks and safeguarding network security. Consequently, it is imperative to seek a new method to differentiate between malicious and normal data within encrypted traffic in real-world network environments.

In the last few years, the application of deep learning technology has expanded significantly and introduced new approaches for the identification of encrypted traffic. Semi-supervised detection methods, which leverage large amounts of unlabeled data, have demonstrated the ability to enhance model accuracy with only a small amount of labeled data in fields such as NLP and CV [4,5]. Thus, the objective of this paper is to detect hidden malicious traffic within encrypted flows, based on features that include network packet sequence characteristics and packet interaction graphs, using only a small amount of labeled normal and malicious traffic. Our study employs a semi-supervised joint training strategy with multimodal fusion decision-making, which exhibits improved detection capabilities for both known and unknown attacks.

### Research Contributions

(1) Extraction of Multimodal Network Traffic Features. Addressing the difficulty in mining deep-level encrypted traffic packet features, we propose multimodal features based on sequence characteristics and heterogeneous graph structures from the perspectives of encryption-independent and transmission interaction behaviors. By analyzing heterogeneous features at different levels, we enhance the model’s robustness against encrypted traffic.
(2) Based on the characteristics of multimodal features, we have designed a unique semi-supervised learning model that combines GRU and GCN. By jointly training the reconstruction error of the autoencoder and the classification loss of the classifier, we aim to improve detection effectiveness. Additionally, we propose an uncertainty estimation of classification results during the training process to better identify unknown malicious traffic.
(3) This paper conducts experimental analysis of our method on two intrusion-detection datasets CICIDS2017 and UNSWNB15, examining various aspects such as different training set label ratios, the presence of unknown attacks, and ablation studies. The results validate the robustness of the proposed model across different scenarios, with the integrated model outperforming any single model.

The second part of the paper investigates the fundamental representations of network traffic and summarizes existing network traffic-detection methods. The third part provides a detailed introduction to our semi-supervised detection method based on multimodal traffic features. The fourth part conducts experiments on two public network-intrusion datasets and analyzes the experimental results. The fifth part concludes the paper.

## 2. Related Work

### 2.1. Extraction of Network Traffic Characteristics

There are numerous methods for extracting network traffic features, which can be categorized into statistical features, payload features, and other features.

Statistical feature extraction typically involves manually designed features such as the number of packets, flow duration, and average packet size. These features are generated by aggregating packet information within the same flow. Barradas et al. [6] proposed a method based on random forests for detecting malicious covert tunnels in multimedia encrypted traffic. This method extracts statistical features based on traffic behavior patterns, including the maximum and minimum packet lengths, the duration and number of packets at peak times, and the number of bytes sent and received for each connection. The study collected normal and malicious multimedia traffic for both QUIC14 and TLS encryption protocols and established a binary classification model using random forests, achieving a 90% detection accuracy for covert tunnels. However, the method has a high false positive rate, exceeding 10%. Anderson et al. [7] presented a method for detecting botnet traffic. It was the first to propose extracting features from TLS handshake information, including low-security cipher suites and self-signed certificates in the certificate content. The method tested various classification algorithms, including random forests, logistic regression, and MLP, all of which achieved high accuracy. However, the detection accuracy for newly discovered malicious traffic was lower than the accuracy achieved in the training set. These methods can effectively reduce data dimensionality, making them more suitable for deep learning tasks. However, they also lose much useful traffic information, such as packet payload and changes in packet behavior over time.

Payload-based feature extraction utilizes the original packet payload content, containing richer traffic information. If the payload representation can be effectively learned, it can address a wider range of attack types. Lin et al. [8] modeled the temporal relationships between bytes and packets in network flows, training a pre-trained model to learn a general vector representation of encrypted traffic. For different detection scenarios, the pre-trained model can significantly accelerate the training of detection models. Kim et al. [9] employed machine learning methods such as K-nearest neighbors and convolutional neural networks to evaluate traffic fingerprinting methods based on Markov chains and other methods. It concludes that, with an appropriate classifier, the Markov chain-based fingerprinting method outperforms other methods in detection effectiveness. Liu et al. [10] introduced a method that constructs multilevel attributes for encrypted traffic based on the distribution of encryption cipher suites and uses these attributes to extract fingerprint information from encrypted traffic.

Currently, alternative methods are beginning to explore the use of sequence features and graph representations based on traffic packets. Typically, only header information is used to construct features, which allows the model to avoid interference from encryption algorithms. Xie et al. [11] presented the HSTF model, a neural network detection model based on spatiotemporal hierarchical features. The model combines CNN and LSTM and takes input data that includes raw image data, packet-level features, and flow-level features. This enhances the model’s ability to learn autonomously and detect HTTP-based malware, achieving an accuracy rate of 99.4%. This method can capture more specific network behavior information than statistical features and can handle encrypted traffic, making it an effective supplement to methods based on statistical features. Rezaei et al. [12] suggested that the order information between adjacent network flows is beneficial for identifying homogeneous flows. They employ LSTM networks to uncover the sequential patterns between network flows.

Graph-based representations are particularly adept at maintaining the topological integrity of network packets. Shen et al. [13] introduced a novel decentralized application fingerprinting method. This method proposes a graph structure named Traffic Interaction Graph, which retains rich original flow characteristics. It uses Multilayer Perceptron (MLP) for vector representation and conducts classification research. However, this method lacks the extraction of flow statistical information and external topological structure information. Busch et al. [14] proposed representing the flow between the same endpoints of network streams as a simple edge and implementing malware detection through graph neural networks.

### 2.2. Malicious Traffic Detection

Based on the types of data covered by the training and test sets, previous detection methods can be primarily categorized into three types.

Supervised detection methods. Experimental results indicate that TLS features, when combined with other features, can achieve higher recall rates. et al. Lee [15] proposed an intrusion-detection method combining a Transformer encoder and an LSTM network. However, supervised detection methods have several limitations. Initially, the prevalence of anomalous instances is significantly less than that of typical instances, which results in suboptimal classification accuracy for the model. Secondly, obtaining label information can be challenging. Thirdly, the model’s generalization cannot be guaranteed to detect unknown attacks.

Unsupervised detection methods. Li et al. [16] presented a method for detecting malicious TLS traffic using clustering techniques. The authors assume that normal TLS traffic is diverse and cannot be clustered into a single class, therefore, outliers in clustering are considered normal flows. On the other hand, traffic originating from the same family of malicious software exhibits similar traits, allowing it to be categorized into unified or multiple classes. Caville et al. [17] tackled the challenge of acquiring high-fidelity, labeled network traffic data in practical settings. They introduced a self-supervised edge embedding technique that combines E-GraphSAGE with advanced graph-based deep learning methods, thereby diminishing the need for labeled samples. This approach leverages the graph neural network algorithm E-GraphSAGE to extract edge attributes and the graph’s topological configuration. Additionally, it implements deep graph mutual information maximization strategies for self-supervised learning processes. Min et al. [18] addressed the issue of imbalanced data in traffic datasets by proposing a dataset balancing method based on Generative Adversarial Networks (GANs). The method processes the UGR’16 dataset to balance the sample distribution, generating attack samples for underrepresented categories using GANs and incorporating them into the original dataset. It then uses a Multi-Layer Perceptron (MLP) neural network to experimentally validate the effectiveness of the balanced dataset. The experimental findings demonstrated that the MLP model is capable of yielding higher precision with the GAN-balanced dataset. However, this method has higher requirements for machine performance and presents challenges in processing non-numeric features such as IP addresses. Unsupervised methods are widely applied due to their ability to adapt well to the variability of network traffic and the absence of manual labeling. However, their accuracy is relatively low.

Semi-supervised detection methods. Semi-supervised methods are less commonly used in network traffic anomaly detection. Sun et al. [19] characterized the semi-supervised context by having an abundance of normal data devoid of anomalies. Their model assimilates the typical patterns exhibited by normal data and contrasts these with the test samples against a normative model to identify potential anomalies. Additionally, Min et al. [20] adhered to a general definition, where only a portion of the training data is labeled, and unlabeled data is used to assist in the learning of labeled data. Wagh et al. [21] employed a self-training method for intrusion-detection tasks. This method labels samples with high prediction confidence from unlabeled data, adds them to the training set, and iteratively refines the process to enhance the training effect of the classifier.

However, most of these methods do not consider the highly imbalanced distribution of normal and anomalous traffic in real-world network environments, nor do they address the detection of unknown attack categories. Their approach is entirely dependent on the intrinsic generalization abilities of the classification model, which retains considerable constraints.

## 3. Method

### 3.1. Preliminaries

#### 3.1.1. Problem Statement

In real-world network environments, it is challenging to collect pure traffic data that is free from noise. Training a detection model with only normal or malicious samples limits its learning capabilities. Therefore, we hypothesize the research scenario: the training dataset comprises a limited set of labeled benign and malicious instances, supplemented by an ample quantity of unlabeled normal samples. The labeled malicious samples may not encompass all types of malicious traffic, indicating that only a subset of malicious categories is known.

#### 3.1.2. Notations

Given N data streams, $x_{1},x_{2},x_{3},\ldots ,x_{N}\in X_{train}$, where M samples have label information, M≪N. ${(x}_{1},y_{1}),{(x}_{2},y_{2}){,(x}_{3},y_{3}),\ldots ,{(x}_{M},y_{M})\in X_{k}\times Y$, and $X_{k}\subseteq X_{train}$, Y={0,1}. We assume that when *y* = 0, the sample belongs to normal traffic, and when y = 1, the sample belongs to malicious traffic. We propose a novel traffic-detection method ψ based on multimodal traffic features, which include network traffic sequence features $T_{s}$ and graph features $T_{g}$. In the test set, we are given T stream data, ${\hat{x}}_{1},{\hat{x}}_{2},{\hat{x}}_{3},\ldots ,{\hat{x}}_{T}\in X_{test}$. We ultimately aim to use the model ψ to provide an anomaly ${score}_{{\hat{x}}_{i}}=\psi \left({\hat{x}}_{i}\right)$ for each stream to assess its likelihood of being malicious traffic.

### 3.2. Framework

**Figure 2:**
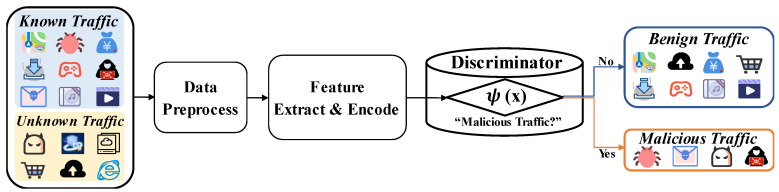
Our semi-supervised approach for the identification of encrypted malicious traffic utilizing features from multiple modalities.

Given that statistical features of network traffic can result in significant loss of information from the original data, we propose a multimodal semi-supervised monitoring model for detecting encrypted malicious traffic. This model is based on traffic sequence analysis and heterogeneous graph embedding. The framework of the model, detailed in Figure 2, encompasses several key components: data preprocessing, feature extraction, semi-supervised learning, and multimodal decision fusion.

### 3.3. Data Preprocessing

The granularity of network traffic detection addressed in this study is focused on the flow level, which typically involves the collection of data packets that share a common set of characteristics, including the source IP, source port, destination IP, and destination port. Since general attacks or abnormal behaviors are often manifested within flows rather than individual packets, this aggregation approach can significantly reduce resource overhead. Compared to unidirectional flows, from the perspective of traffic interaction, bidirectional flows with richer information are chosen as the detection granularity. Therefore, we categorize the packets in PCAP files into different session flows based on the five-tuple, which facilitates subsequent labeling and processing. The segmentation process utilizes the tool pkt2flow [22], using source address, destination address, source port, and destination port as classification information to split the packets into bidirectional session flows, with each session flow saved to a separate PCAP file. Subsequently, invalid packets are removed, and dirty data within each session flow after segmentation, including ARP packets, DNS packets, and retransmission packets, are cleared to minimize their impact on the detection results.

### 3.4. Multimodal Feature Extraction and Encoding

#### 3.4.1. Sequence Feature

We use only the header portion of each traffic data packet for feature extraction. The features extracted for each packet are listed in Table 1. In total, we extract five types of packet features, which effectively reflect the unique properties of traffic data packets within the sequence.

Among these, the target port number does not inherently possess a measure of magnitude and is not suitable for direct treatment as a continuous feature. Consequently, we consider encoding it as a discrete value. The range of port number features is extensive, potentially spanning the interval [0, 65536]. Therefore, using One-Hot Encoding is impractical. As a result, we have resorted to binary encoding for all features except for time intervals and packet payload sizes. We also utilize the size of the packet payload and the size of the packet header as features. Since the header size can indicate the type of multi-layer protocol to some extent, we consider encoding it as a discrete value. The numerical value of the payload size is more meaningful, so we directly use numerical encoding for it. Compared to traditional statistical features, our method based on packet sequence modeling can capture more detailed patterns of traffic behavior, effectively leveraging the temporal attributes of packets. Additionally, due to the low computational cost of feature extraction, this approach offers significant advantages in terms of time efficiency.

We utilize an autoencoder model based on Gated Recurrent Units (GRU) to extract and reconstruct features from unlabeled sequence data. GRUs are effective in encoding variable-length input sequences into fixed-length vectors. Let $s_{i}$ represent the current input sample sequence, $s_{i}=\left(e_{1},e_{2},\ldots ,e_{n}\right)$ and n denote the length of sequence $s_{i}$. Consequently, the relationship for the hidden state of the first GRU layer can be expressed as shown in Equation (1).

(1) $$h_{t}^{\left(1\right)}=\left\{\begin{array}{c} 0,\;t=0\\ GRU\left(h_{t-1}^{\left(1\right)},e_{t}\right)\;,\;1\leq t\leq n \end{array}\right.$$

$h_{t-1}$ represents the hidden state from the previous moment. The GRU() function denotes the transition relationship between hidden states at adjacent moments. We employ a stacked multi-layer GRU architecture for encoding, with a total of H  layers. For layers where *i* > 1, the input to the ith GRU unit is generated by the output of the previous layer at the same time step. Since the GRU uses the hidden state G for information transfer between adjacent moments and directly for the output of the GRU unit at that moment, the relationship for the i-th layer GRU can be expressed as Equation (2).

(2) $$h_{t}^{\left(i\right)}=GRU(h_{t-1}^{\left(i\right)},h_{t}^{\left(i-1\right)}),\;1\leq t\leq n,\;i>1$$

Essentially, we utilize the output of the hidden state from the last layer as a fixed-length vector representation z that we obtain from the encoding process, which can be denoted as $z=h_{n}^{\left(H\right)}$.

Similarly, the decoder is composed of several layers of GRU units and a dense layer, configured to calculate the discrepancy in the reconstruction between the generated sequence and the original input. Within the decoder, the ultimate output z derived from the encoder serves as the initial input, as demonstrated in Equation (3).

(3) $${\hat{h}}_{t}^{\left(i\right)}=GRU({\hat{h}}_{t-1}^{\left(i\right)},z),\;1\leq t\leq n$$

**Figure 3:**
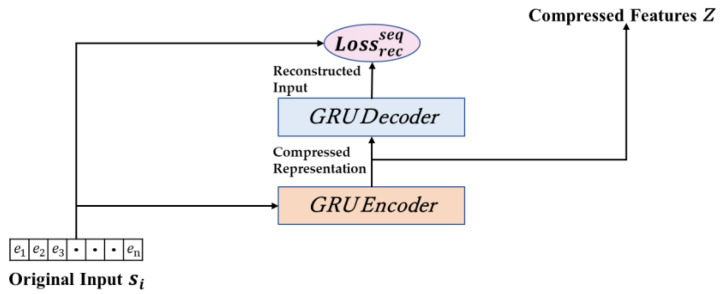
Illustration of traffic sequence feature extraction.

At the final stage of the decoder, we employ a fully connected layer to generate the reconstructed sequence, which is then employed to calculate the reconstruction loss $L_{rec}^{seq}$, as illustrated in Equation (4).

(4) $$L_{rec}^{seq}=\sum_{i=1}^{N}\sum_{t=1}^{n_{i}}l(e_{t}^{\prime i},e_{t}^{i})$$

l represents the distance function between vectors, and we use the mean squared error for this calculation. Figure 3 depicts the process of extracting traffic sequence features and calculating reconstruction loss.

#### 3.4.2. Graph Feature

**Figure 4:**
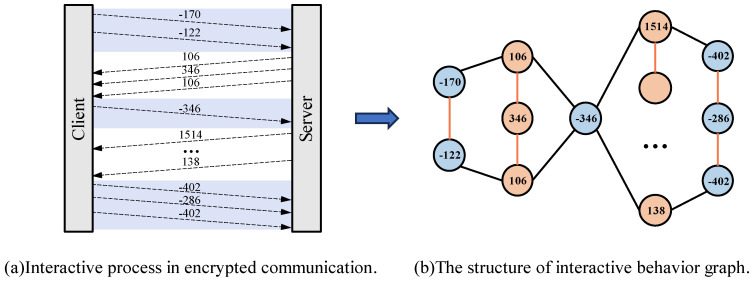
Illustration of traffic graph feature construction. The transfer of packets between client and server is transformed into a heterogeneous graph representation.

To avoid feature overlap between traffic flows of different categories and versions, we describe the traffic features from another perspective as a supplement to flow sequence features. This topological feature can capture the connection behaviors between different host applications. Moreover, the low-dimensional feature vectors generated through graph embedding technology are independent of the statistical features of encrypted flow data and possess a certain level of anti-interference capability. Building upon the work [13], we further optimized the traffic interaction graph by embedding packet size, direction, order, and graph-level packet information into the heterogeneous graph neural network. We define the packet length as the vertices of the graph structure and arrange them in sequence within the graph. To represent the direction of packets in the graph structure, we use positive and negative signs to denote downstream and upstream packets, respectively. Subsequently, the entire flow is divided into multiple burst data packets based on the packet-transmission direction. First, we connect the vertices within the same group of burst data packets with edges. Unlike [13], we use a different type of edge to connect different groups of burst data packets in sequence. Figure 4 provides a detailed illustration of the heterogeneous traffic burst graph. Different colored lines in the figure represent two types of edges.

**Figure 5:**
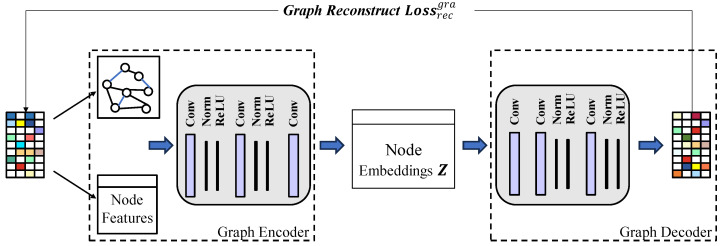
Framework of graph encoder network.

We employ a heterogeneous graph encoder that combines different relationships within the heterogeneous graph to learn high-quality representations of network traffic. The strength of a heterogeneous graph is its capacity to encapsulate diverse types of relationship data, effectively converting high-dimensional, sparsely distributed topological graph data into concise vector forms. This transformation aims to preserve the structural and semantic details of the nodes to the greatest extent possible. As depicted in Figure 5, we deploy a GCN to distill the representation for each node within the graph. The convolutional layer operation in GCN can be formalized as Equation (5).

(5) $$H^{\left(l+1\right)}=\sigma ({\tilde{D}}^{-\frac{1}{2}}\tilde{A}{\tilde{D}}^{-\frac{1}{2}}H^{\left(j\right)}W^{\left(j\right)})$$

where, $\tilde{D}=D+I_{N},\;\;\tilde{A}=A+I_{N},\;I_{N}$ represents the identity matrix. D represents the degree matrix of the nodes, which indicates the degree of each node. A represents the adjacency matrix. $H^{\left(j\right)}$ represents the feature matrix of the j-th layer. $W^{\left(j\right)}$ represents the learned weights. σ denotes the non-linear activation function. After encoding through the GCN layer, the final embedded feature representation is given by Equation (6).

(6) $$Z=GCN\left(GCN(X,A)\right)=softmax({\tilde{D}}^{-\frac{1}{2}}\tilde{A}{\tilde{D}}^{-\frac{1}{2}}\cdot \sigma ({\tilde{D}}^{-\frac{1}{2}}\tilde{A}{\tilde{D}}^{-\frac{1}{2}}XW^{\left(0\right)})W^{\left(1\right)})$$

The decoder part of the graph convolution typically computes the reconstructed adjacency matrix $\hat{A}$ through the use of an inner product, as indicated in Equation (7).

(7) $$\hat{A}=\sigma \left(ZZ^{T}\right)=sigmoid\left(ZZ^{T}\right)$$

Throughout the training phase, the objective of the decoder is to reduce the discrepancy between the original graph dataset and the reconstructed version, thereby enabling the acquisition of robust feature representations. Commonly, the graph reconstruction error is quantified using the mean squared error (MSE) as the primary metric for the overall graph reconstruction loss, as indicated in Equation (8).

(8) $$L_{rec}^{gra}=\sum_{i=0}^{N}MSE(A,\hat{A})$$

### 3.5. Semi-Supervised Co-Training

**Figure 6:**
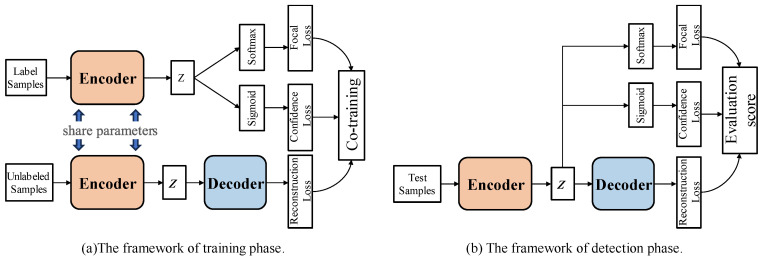
Description of our semi-supervised co-training framework. The process primarily consists of two components: the training phase and the detection phase.

Given that the training dataset $X_{train}$ comprises a limited set of labeled instances and a substantial amount of unlabeled ones, traditional semi-supervised classification methods often utilize all unlabeled data for pre-training, training an autoencoder network, and learning reconstruction error loss to better learn the original features and reduce dimensionality. Subsequently, one can continue to fine-tune the original encoder using labeled data to train the final classifier. However, we found that the method of initializing the classifier weights through pre-training often encounters performance limitations. Therefore, we design a joint training approach for the autoencoder and the classifier, simultaneously learning reconstruction error and classification error to update the model weights. In this context, the autoencoder acts as a regularizer, which often leads to better performance than the general separate model. The specific training phase process is depicted in Figure 6. The model can be divided into the training phase and the detection phase.

The classifier module first utilizes the output of the encoder as the supervised learning input for labeled data ${\tilde{s}}_{i}$, which, after being encoded by the encoding layer, yields a fixed-length vector ${\tilde{o}}_{i}$. We input the ${\tilde{o}}_{i}$ into a fully connected layer and a softmax layer to train a binary classifier, with the output representing the predicted probability ${\tilde{p}}_{i}$ of the classification result.

To counteract the challenge of a significant imbalance between the positive and negative samples in the training data, we employ the focal loss [23], function in place of the standard cross-entropy loss. This specialized loss function diminishes the impact of a multitude of straightforward negative examples during training, thereby boosting the model’s capacity to learn from complex samples. The computation of this loss is depicted in Equation (9).

(9) $$FL({\tilde{p}}_{i})=-\alpha {\left(1-{\tilde{p}}_{i}\right)}^{\gamma }\cdot log({\tilde{p}}_{i})$$

α and γ are weighting factors that respectively control issues of sample imbalance and the difficulty of recognition. We have adopted the value of 2 for γ as recommended in the focal loss [23], and we have used the default value of 1 for α.

For the detection of unknown malicious traffic, we concurrently design a confidence-estimation method during the training of the classifier. When faced with unknown samples, the probability output of a typical classifier cannot directly represent the model’s confidence in the classification result. Even if a sample does not belong to the same distribution as the training set of the classifier, the classifier often provides a very high probability output, even for an incorrect prediction. Therefore, we introduced an uncertainty-estimation method in the classification module to calculate the confidence ${\tilde{c}}_{i}$ estimate for samples in the test set. The architecture primarily comprises several dense layers and a sigmoid activation function. The confidence score is bounded within the interval [0, 1], where a higher score indicates higher certainty. Facing unknown anomalies in the test set often results in lower confidence, which will help improve the model’s ability to detect unknown anomalies. To incorporate confidence into the model training, we adjust the classification prediction probabilities as indicated in Equation (10).

(10) $${\tilde{p}}_{i}^{\prime }=\left\{\begin{array}{c} {\tilde{c}}_{i}\cdot {\tilde{p}}_{i}^{1}{\tilde{y}}_{i}=0\\ {\tilde{c}}_{i}\cdot {\tilde{p}}_{i}^{1}+(1-{\tilde{c}}_{i}){\tilde{y}}_{i}=1 \end{array}\right.$$

${\tilde{p}}_{i}^{\prime }$ represents the probability that a predicted sample is malicious, and similarly, the probability that a sample is benign is ${1-\tilde{p}}_{i}^{\prime }$. Intuitively, when the predicted confidence is high, we use the original prediction to calculate the loss, while when the confidence is low, the training provides a hint, i.e., we calculate the loss using the true label.

During the training phase, a joint training process is achieved through alternating labeled and unlabeled data. The ultimate loss function is a combination of the classification loss and the reconstruction loss, as shown in Equation (11).

(11) $$L=L_{rec}+\mu L_{cls}$$

where μ is used to balance the classification loss and reconstruction loss.

Figure 6b represents the anomaly-detection stage, where we combine three loss functions that are meaningful for detecting unknown malicious traffic samples to evaluate anomalies. For each test sample ${\hat{x}}_{i}$, we calculate its anomaly score ${score}_{{\hat{x}}_{i}}$, as shown in Equation (12).

(12) $${score}_{{\hat{x}}_{i}}=\lambda \cdot {score}_{cls}^{i}\cdot {score}_{conf}^{i}-\theta \cdot {score}_{conf}^{i}+{score}_{rec}^{i}$$

We aim to jointly determine the anomaly score based on the classification results from supervised learning and the reconstruction error from unsupervised learning. We use the product of the probability of classifying a sample as anomalous and the model’s confidence as the core influence of the classifier, with λ being used to adjust the weights. Additionally, we consider the possibility of classifying a sample as an unknown malicious type when the confidence is low. Therefore, we also use a penalty term θ to assign a higher anomaly estimate to samples with low confidence and sum it with the reconstruction error to obtain the final anomaly score. In summary, for samples with high confidence in the classification results, the model tends to rely on supervised learning outcomes for judgment. Conversely, for samples with low classification confidence, there is a strong likelihood that they belong to unknown categories, and we prefer to use reconstruction error for determination.

### 3.6. Multimodal Fusion

In this section, we describe the detection framework based on multimodal data. For each flow, we can obtain two types of features: traffic sequence features and heterogeneous graph embedding features. We aim to adopt an appropriate multimodal strategy to effectively combine the multimodal features of network traffic. To effectively utilize the two distinct feature representations of sequences and topological graphs, and to integrate with the semi-supervised joint training model proposed by us, we will construct a multimodal detection framework using a post-processing fusion approach, which is also known as decision fusion.

**Figure 7:**
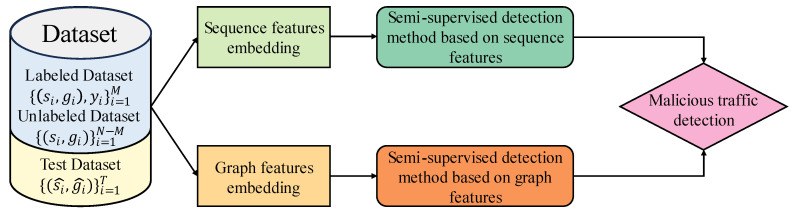
Overall discriminator model based on multimodal data.

As shown in Figure 7, we train a semi-supervised model using an unlabeled dataset and a labeled dataset. The sequence features $s_{i}$ and the heterogeneous graph features $g_{i}$ are used to train these two models, respectively. For the test sample, we combine the ${\mathrm{score}}_{\mathrm{total}}$ output by the two models to measure the probability that the sample belongs to malicious traffic. The parameter τ is used to adjust the weights of the two models.

(13) $${\mathrm{score}}_{\mathrm{total}}={\;\mathrm{score}}_{\mathrm{seq}}+{\tau \cdot \;\mathrm{score}}_{\mathrm{graph}}$$

## 4. Experiment

### 4.1. Dataset

To substantiate the robustness of our suggested model in various settings, we performed experiments utilizing two publicly available datasets, namely UNSW-NB15 [24] and CICIDS2017 [25].

**Figure 8:**
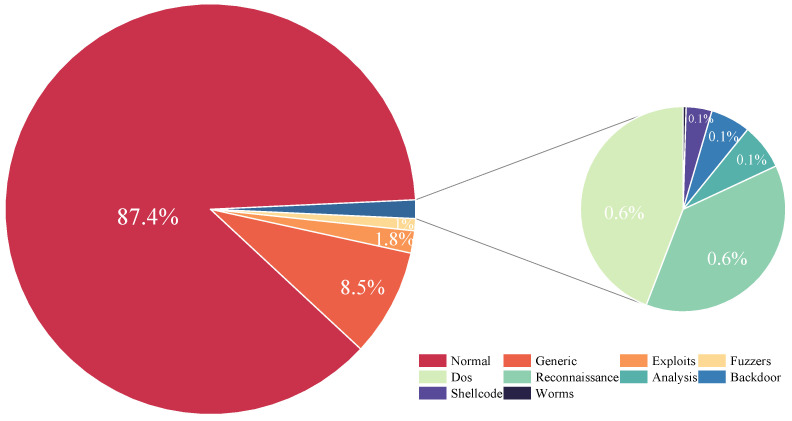
Overall category distribution of the UNSW-NB15.

**UNSW-NB15** is a network attack dataset that includes genuine network traffic and composite malicious communication behaviors within a real network environment. The records are primarily categorized into benign and malicious types, with the malicious records further divided into nine categories. The original network data in the dataset was created by the Cyber Range Lab, utilizing the IXIA PerfectStorm tool (https://research.unsw.edu.au/projects/unsw-nb15-dataset, accessed on 8 October 2024). The quantities of each category are listed in Table 2 and Figure 8. In the UNSW-NB15 dataset, the majority of the data is normal traffic, with Generic and Exploits attack types also accounting for a significant proportion. The execution complexity of various attack behaviors, the potential benefits, and their occurrence frequency result in a highly skewed distribution of attack types within the network traffic data. This imbalance presents a considerable challenge for the detection of anomalous network traffic patterns.

**Figure 9:**
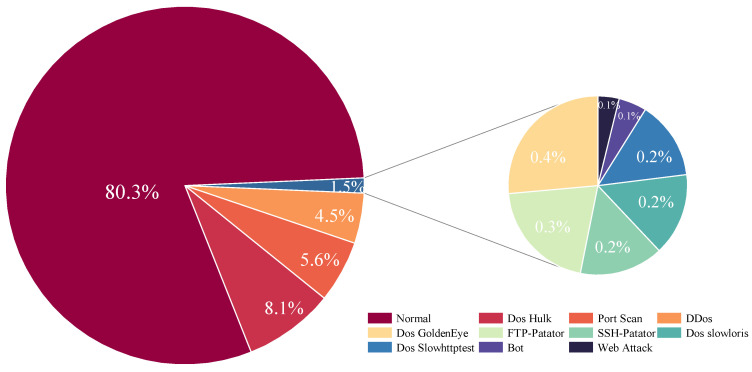
Overall category distribution of the CICIDS2017.

**CICIDS2017** collects five days of simulated real-world traffic generated by the B-Profile system, encompassing a variety of network services and attack methods, including DoS, DDoS, and Port Scanning, among others. Consequently, the dataset contains both benign and various malicious traffic, widely used for the classification of encrypted traffic. This paper filters out 10 types of attacks, with the specific categories and their quantities listed in Table 3. Figure 9 presents a pie chart that graphically depicts the categorical distribution within the dataset.

### 4.2. Environment and Parameters Setting

The experiments were conducted on a Windows 10 system with Python 3.7 and PyTorch 1.10. During the preprocessing stage of the traffic data, the open-source tool pkt2flow is used to convert the original PCAP files into session flows. In the stages of model construction and training, the model was built and trained using PyTorch, with optimization performed on an NVIDIA GeForce RTX 4080 for acceleration. For the sequential feature encoder, the GRU model can easily perform reconstruction. Thus, we equate the classification loss with the reconstruction loss, setting μ to 1. For the graph feature encoder, the RGAT model experiences a higher reconstruction loss during the initial epochs of iteration. To balance the joint training loss, we set μ to 2 for the graph structure branch. To standardize the units, we manually set λ to 1 and θ to 0.05. Table 4 presents the parameter values for the network structure attributes chosen in our model.

### 4.3. Evaluation and Validation Metrics

This section utilizes four key performance indicators for assessment: accuracy, precision, recall, and the F1 score. Accuracy, the most frequently used metric, indicates the ratio of correctly identified instances to the total instances. Precision is defined as the ratio of true positive instances to the total number of instances labeled as positive. Recall, on the other hand, is the ratio of true positive instances to the total actual positive instances. The F1 score is a metric that provides a balance between precision and recall. To reduce the variability from a single trial, this study conducts the experiment five times, each with a random split of the dataset into training and testing subsets. The final experimental result is determined by averaging the results from these five iterations.

We design three sets of experiments to evaluate the performance of various methods:

(1) Performance of the model under different label ratios.

During the semi-supervised training phase, different quantities of labeled samples were used to train each model to observe the performance under varying degrees of labeled samples.

(2) Performance in detecting unknown malicious traffic.

For the two datasets, the experimental scenario was uniformly set to 1% label ratio. Two types of malicious traffic are selected from each dataset as unknown categories for experiments, and corresponding ROC curves were plotted to observe the detection results.

(3) The role of each component in our model.

To analyze the actual effects of each component in the model, including confidence estimation, reconstruction error, and the effect of multimodal fusion. Experiments were conducted under a known label ratio of 1% in the IDS2017 dataset.

To more thoroughly assess the efficacy of our proposed model, we select various baselines for comparison. An introduction to the selected methods is as follows.

- CNN+LSTM Model [11]: This model combines convolutional neural networks and long short-term memory to integrate spatial and temporal features of traffic data.
- E-GraphSAGE Model [18]: This model is the first to introduce graph neural networks into the field of anomaly traffic detection, focusing on the aggregation and update of graph information on edge features.
- E-ResGAT Model [26]: This model replaces the backbone network with Graph Attention Networks on the basis of E-GraphSAGE, introduces attention mechanisms into graph neural networks, calculates the mutual importance of each node to its adjacent nodes, and participates in the aggregation of information on the graph.
- BYOL-NIDS Model [27]: This model implements self-supervised learning for anomaly traffic-intrusion detection, introduces the BYOL model into the field of anomaly traffic detection and specifically performs data augmentation.
- RUIDS Model [28]: This model implements an anomaly traffic-detection scheme based on self-supervised masked time context using the Transform.
- VGAE Model [29]: This model uses variational graph auto-encoders to achieve self-supervised capture and learning of feature representations of traffic graphs for traffic classification.
- CNN+AE [30]: Composed of a pre-trained CNN model and an autoencoder model for classification. The CNN model automatically extracts deep features from the payload of network traffic, and the AE model judges normal and malicious traffic through the reconstruction error of deep features.

### 4.4. Detection Analysis on Known Samples and Varying Label Ratios

In the experiment with known sample verification, both the training and test data consist of the same malicious family. The objective of this experiment is to verify whether different detection models can effectively distinguish between malicious and normal traffic. Table 5 first presents the detection results of our proposed model and other baseline models on the complete labeled training set.

On the UNSW-NB15 dataset, our model achieves the Acc, Pre, Rec, and F1 rates by 99.51%, 99.46%, 99.36% and 99.41% respectively. It can be observed that on the IDS2017 dataset, our model still achieves the best classification performance. All four metrics have exceeded 99%. This demonstrates that our model has good generalization capabilities. The E-ResGAT model, which employs an attention mechanism to focus on the channel dimensions of traffic features, yields good results.

In real network environments, malicious data is far less common than benign data. To better reflect this characteristic in dataset validation. We set up training sets with different proportions of labeled malicious traffic. For each of the two datasets, we will evaluate whether the model can still achieve good performance with a very small amount of labeled data. We will conduct experiments with label proportions in the training set at 0.5%, 1%, 5%, 10%, and 20%. The F1 score on the CICIDS2017 dataset, as shown in Table 6, indicates that the method proposed by us consistently achieves nearly the best performance across varying label ratios. When the percentage of labeled training samples exceeds 10%, all methods exhibit similar performance to the previous experiment. The performance of all other models, except for ours, has significantly decreased to varying degrees when the label ratio is low. Specifically, when the labeling rate is reduced to 5%, our method outperforms current baseline methods by more than 2.69%. As the labeling rate decreases to 1% and 0.5%, supervised learning models [11,18,26] tend to focus more on extracting features from normal traffic. The insufficient extraction of malicious traffic features often leads to a significant decrease in the F1 score. When compared to self-supervised learning methods [27,28,29], our approach demonstrates significant advantages, outperforming the best models by 3.49% and 5.69% in F1 score, respectively. We attribute this to our joint training method and confidence-estimation design, which more effectively utilizes unlabeled data to enhance the model’s robustness.

**Figure 10:**
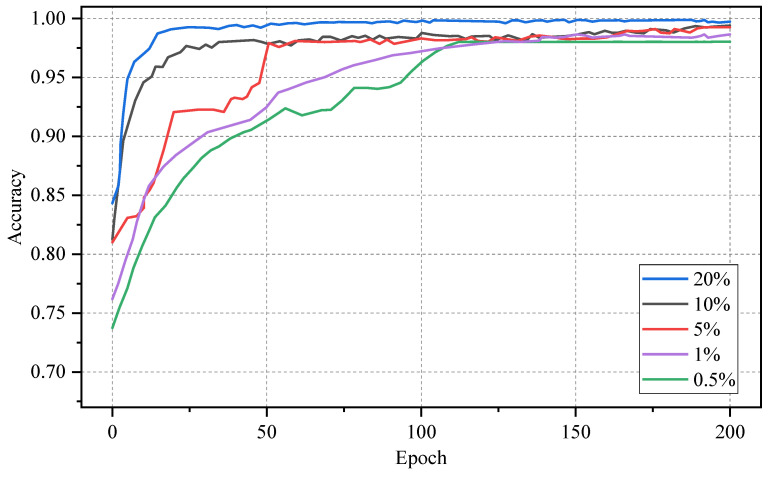
Accuracy curves of our model during training with different label ratios.

Figure 10 illustrates the accuracy rates of our model during each epoch of training on CICIDS2017, with labeled samples comprising 0.5%, 1%, 5%, and 10% of the dataset. The performance of our model is slightly influenced by the number of labeled samples in the early stages. As the number of iterations increases, the accuracy of our method rapidly improves, compensating for the insufficient number of training samples. After 150 iterations, the model trained with 1% labeled samples has achieved the same performance as the one trained with 10% labeled samples. However, both methods still exhibit a performance gap compared to the model trained with 20% labeled samples.

### 4.5. Detection Analysis on Unknown Attacks

In this part, we primarily evaluate the model’s detection effectiveness for attacks of unknown categories. In the experimental process, we start with the initial data partition that excludes unknown attacks. We remove all samples corresponding to the attack categories from the labeled training data, while still retaining these samples in the unlabeled data and test sets. Equally, we have standardized the experimental setup to a 1% labeled data ratio. We conduct a comparative study of our method with four baseline models that have shown good performance in known attack detection. Furthermore, to mitigate the impact of training instability on the assessment, we performed five repetitions for each experimental scenario and calculated the average results.

In selecting samples from unknown categories, we aim to train the detector using common and low-risk attack traffic to identify those categories that are high-risk and highly stealthy. Consequently, for the UNSW-NB15 dataset, we have selected Backdoor and Exploits attack traffic for the test set. For the CICIDS.2017 dataset, we have chosen PortScan and DoS Hulk attack traffic to be included in the test set.

**Figure 11:**
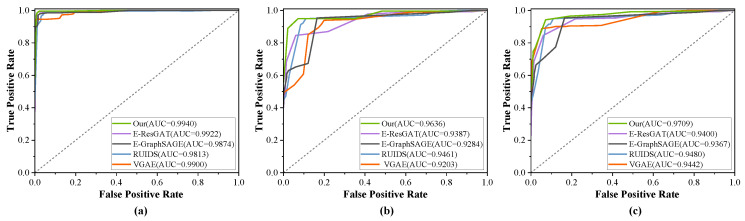
Evaluation of unknown attack detection on the UNSW-NB15 dataset. (**a**) Without unknown attacks. (**b**) With unknown attacks as backdoor. (**c**) With unknown attacks as exploits.

**Figure 12:**
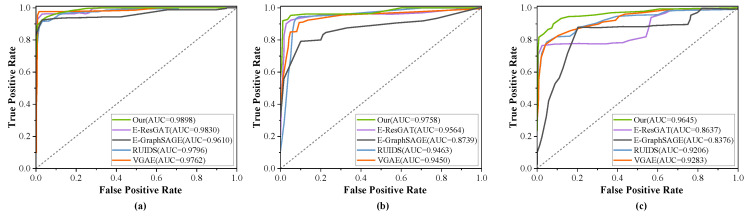
Evaluation of unknown attack detection on the CICIDS2017 dataset. (**a**) Without unknown attacks. (**b**) With unknown attacks as DoS Hulk. (**c**) With unknown attacks as PortScan.

Figure 11 and Figure 12 correspond to the ROC curves for three scenarios in the UNSW-NB15 and CICIDS2017 datasets, respectively. It is observable that the detection efficacy of methods relying on classifiers markedly diminishes in the presence of unknown attacks. With the exception of our method, the other methods have weak capabilities in identifying unknown attacks. Methods [18,26] based on supervised learning have a high rate of misclassification, confirming that they are insufficient in characterizing traffic interaction behaviors. In contrast, the detection effectiveness of semi-supervised methods experiences only a slight reduction. However, our method, which is based on multimodal feature extraction and semi-supervised training, shows no significant change. The embedding features calculated by our method are not confined to the characteristics of a single network session, but rather represent the multidimensional features of network traffic, which can uncover more classification features for both benign and malicious traffic. Therefore, the method introduced in this chapter is better suited to detect unknown attack traffic.

### 4.6. Ablation Experiment

#### 4.6.1. Effect Analysis of Model Components

Finally, we analyze the practical effects of each component of our model. This includes an analysis of the effectiveness of confidence estimation, reconstruction error, different classification loss functions, and single-modal models. For the following experiments, we set the experimental scenario to have a label ratio of 1%.

The ablation study results are presented in Table 7. Compared to the original model proposed in this paper, the detection performance of all variants, which have had one module removed, has decreased to varying degrees. This indicates that each module plays a positive role in the detection of anomalous traffic. The removal of the GCN component results in a significant decrease in model performance, indicating that graph neural networks are more effective at extracting correlations between features, thereby detecting more subtle attacks. It can be observed that the F1 score significantly decreases when the focal loss function is substituted by cross entropy loss, indicating that the model may have reduced its ability to detect covert classes.

#### 4.6.2. Parameter Sensitivity Analysis

**Figure 13:**
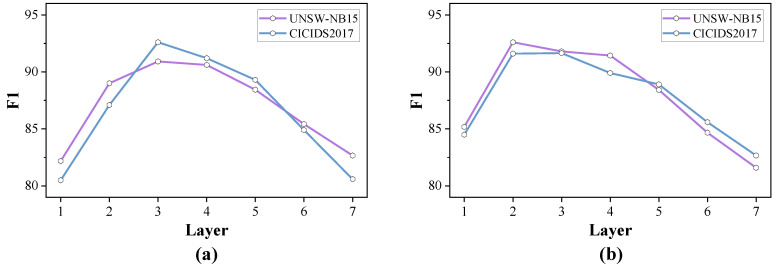
F1 Scores for models with different layer configurations. (**a**) GRU layers. (**b**) GCN layers.

We conducted a parameter comparison between the number of GRU layers and GCN layers in semi-supervised learning, examining the impact of varying network layer depths on model accuracy, as shown in Figure 13. By stacking layers in the graph neural network, the model can capture higher-order neighbor feature information, leading to a more precise representation. However, an excessive number of layers can result in over-smoothing, where the features of different nodes become homogenized, making it difficult to distinguish between nodes and negatively impacting model performance. Consequently, the model’s accuracy initially increases with the addition of layers but eventually decreases. According to the experimental data, configuring the model in this study with 3 GRU layers and 2 GCN layers strikes a balance that efficiently captures the characteristics of network flows.

Additionally, we assess the importance of the branches for the fusion of sequential and graph features. We assign three different values to τ to combine them, and the classification results are shown in Table 8. The results indicate that different settings yield satisfactory outcomes, demonstrating that both sequential features and graph structures are equally important for the identification of malicious traffic. Therefore, in our experiment, we assign equal decision weights to both modalities.

## 5. Conclusions

To address the challenges in malicious traffic detection, such as the difficulty in labeling samples and capturing attack traffic, as well as the limitations of existing deep learning methods in uncovering subtle malicious activities and low detection accuracy, this paper introduces a multimodal approach for detecting malicious traffic. We characterize encrypted traffic by leveraging both sequential and graph structural features of the traffic flow. Initially, we segment the traffic based on session granularity and then train a multimodal deep learning model that integrates these two types of features, providing a holistic description of malicious traffic behavior. The co-training of confidence loss and classification loss effectively mitigates the issue of insufficient sample information mining common in existing semi-supervised training methods, enhancing the robustness of the classifier. Experimental results on multiple datasets demonstrate the effectiveness of the proposed fusion method for malicious network traffic detection, particularly outperforming benchmark methods in scenarios with scarce labeled samples and the discovery of unknown malicious categories.

To advance the research on encrypted malicious traffic detection, future efforts will concentrate on the following areas: (1) Enhancing the scale of samples. It is well recognized that the quantity of samples significantly impacts deep learning models; hence, we will explore the use of contrastive representation learning ang generative adversarial networks to augment the sample size. (2) Improving the interpretability of traffic features. While deep learning is powerful in its end-to-end data learning capabilities, its interpretability has been a persistent challenge. Poor interpretability can limit the practical application of traffic features.

## Figures and Tables

**Table 1:** Overview of traffic sequence characteristics.

Feature	Coded Format
Destination Port	Binary coding
Time Interval	Numerical coding
Packet Header Size (Byte)	Binary coding
Packet Payload Size (Byte)	Numerical coding
TCP Window Size	Binary coding

**Table 2:** The composition of the UNSW-NB15 dataset.

Classes	Quantity
Normal	2,218,761
Reconnaissance	13,987
Worms	174
Dos	16,353
Generic	215,481
Analysis	2677
Fuzzers	24,246
Shellcode	1511
Backdoor	2329
Exploits	44,525
**Total**	**2,540,044**

**Table 3:** The composition of the CICIDS2017 dataset.

Classes	Quantity
Normal	2,271,320
Dos Hulk	230,124
Port Scan	158,804
DDos	128,025
Dos GoldenEye	10,293
FTP-Patator	7935
SSH-Patator	5897
Dos slowloris	5796
Dos Slowhttptest	5499
Bot	1956
Web Attack	1507
**Total**	**2,827,156**

**Table 4:** The main parameters setting.

Parameters	Default Value
Epoch	200
Learning rate	0.0005
Optimizer	Adam
Batch size	64
Layer of GRU	3
Layer of GCN	2
α, γ	(1, 2)
μ	1, 2
λ,θ	(1, 0.05)
*τ*	1

**Table 5:** Comparison of detection performance on UNSW-NB15 and CICIDS2017.

Model	UNSW-NB15				CICIDS2017			
	Acc	Pre	Rec	F1	Acc	Pre	Rec	F1
1D-CNN	96.61	96.55	96.51	96.53	95.16	91.63	95.39	89.79
LSTM	95.10	92.94	95.17	91.83	96.78	96.07	96.56	95.83
CNN+LSTM	97.95	97.03	97.48	96.80	98.02	98.05	98.20	98.25
E-GraphSAGE	98.93	98.64	98.18	98.41	99.09	99.06	99.29	98.94
E-ResGAT	99.40	99.50	98.90	99.20	99.84	99.65	99.43	99.76
BYOL-NIDS	97.05	97.26	98.72	96.54	96.70	95.24	95.74	94.99
RUIDS	98.91	98.80	98.86	98.77	99.09	99.06	99.69	98.75
VGAE	98.63	98.80	97.63	99.38	97.20	97.26	97.40	97.19
CNN+AE	96.96	97.19	97.22	97.17	98.64	98.62	98.59	98.63
Our	99.51	99.46	99.36	99.41	99.99	99.98	99.98	99.98

**Table 6:** Comparative experiment on different label ratios on CICIDS2017.

	0.5%	1%	5%	10%	20%
1D-CNN	67.81	81.09	88.61	90.55	94.36
LSTM	70.95	79.23	89.08	92.32	96.09
CNN+LSTM	78.18	82.71	90.16	92.80	97.81
E-GraphSAGE	80.78	92.72	94.86	95.77	98.56
E-ResGAT	82.02	94.10	95.74	96.79	99.13
BYOL-NIDS	85.29	90.73	92.72	95.09	96.07
RUIDS	92.33	95.16	96.71	97.68	98.84
VGAE	87.05	93.72	96.44	96.81	97.15
CNN+AE	79.59	84.71	93.16	94.66	98.41
Our	98.02	98.65	99.40	99.40	99.73

**Table 7:** Ablation study of key components on the UNSW-NB15 and CICIDS2017 dataset.

Dataset	Method	Acc	Pre	Rec	F1
UNSW-NB15	w/o confidence estimation	95.91	95.28	95.36	95.32
	w/o reconstruction error	94.84	93.67	93.22	93.44
	w/o sequence feature	91.29	91.40	90.25	90.82
	w/o graph feature	90.89	90.46	90.99	90.72
	w/cross-entropy loss	94.96	94.28	93.33	93.80
	default (all)	97.63	98.37	94.23	96.26
CICIDS2017	w/o confidence estimation	97.42	97.36	96.74	97.05
	w/o reconstruction error	96.55	95.23	94.55	94.89
	w/o sequence feature	94.63	93.74	93.56	93.65
	w/o graph feature	94.19	93.80	93.93	93.86
	w/cross-entropy loss	96.32	96.95	95.29	96.11
	default (all)	98.79	98.68	98.63	98.65

**Table 8:** Ablation study of key parameter on the UNSW-NB15 and CICIDS2017 dataset.

	τ	Acc	Pre	Rec	F1
UNSW-NB15	0.5	97.67	97.70	94.66	96.16
	1	97.63	98.37	94.23	96.26
	5	97.46	96.84	94.57	95.69
CICIDS2017	0.5	98.67	98.85	98.58	98.71
	1	98.79	98.68	98.63	98.65
	5	97.84	98.13	98.40	98.26

## Data Availability

The data presented in this study are openly available in [24,25].
